# Polycaprolactone for Hard Tissue Regeneration: Scaffold Design and In Vivo Implications

**DOI:** 10.3390/bioengineering12010046

**Published:** 2025-01-08

**Authors:** Fernanda Ramírez-Ruiz, Israel Núñez-Tapia, María Cristina Piña-Barba, Marco Antonio Alvarez-Pérez, Vincenzo Guarino, Janeth Serrano-Bello

**Affiliations:** 1 Tissue Bioengineering Laboratory, Division of Graduate Studies and Research, Faculty of Dentistry, National Autonomous University of Mexico, Circuito Exterior s/n, University City, Coyoacán, Mexico City 04510, Mexico; mramirezruiz957@gmail.com (F.R.-R.); malva@fo.odonto.unam.mx (M.A.A.-P.); 2 Materials Research Institute, National Autonomous University of Mexico, Circuito Exterior s/n, University City, Coyoacán, Mexico City 04510, Mexico; isrant86@gmail.com (I.N.-T.); crispina99@gmail.com (M.C.P.-B.); 3 Institute of Polymers, Composite and Biomaterials, National Research Council of Italy, Mostra d’Oltremare, Pad 20, V.le J.F.Kennedy 54, 80125 Naples, Italy

**Keywords:** biomaterials, synthetic polymer, polycaprolactone, tissue engineering, scaffolds, bone tissue, PCL in vivo implications

## Abstract

In the last thirty years, tissue engineering (TI) has emerged as an alternative method to regenerate tissues and organs and restore their function by implanting specific lineage cells, growth factors, or biomolecules functionalizing a matrix scaffold. Recently, several pathologies have led to bone loss or damage, such as malformations, bone resorption associated with benign or malignant tumors, periodontal disease, traumas, and others in which a discontinuity in tissue integrity is observed. Bone tissue is characterized by different stiffness, mechanical traction, and compression resistance as a function of the different compartments, which can influence susceptibility to injury or destruction. For this reason, research into repairing bone defects began several years ago to find a scaffold to improve bone regeneration. Different techniques can be used to manufacture 3D scaffolds for bone tissue regeneration based on optimizing reproducible scaffolds with a controlled hierarchical porous structure like the extracellular matrix of bone. Additionally, the scaffolds synthesized can facilitate the inclusion of bone or mesenchymal stem cells with growth factors that improve bone osteogenesis, recruiting new cells for the neighborhood to generate an optimal environment for tissue regeneration. In this review, current state-of-the-art scaffold manufacturing based on the use of polycaprolactone (PCL) as a biomaterial for bone tissue regeneration will be described by reporting relevant studies focusing on processing techniques, from traditional—i.e., freeze casting, thermally induced phase separation, gas foaming, solvent casting, and particle leaching—to more recent approaches, such as 3D additive manufacturing (i.e., 3D printing/bioprinting, electrofluid dynamics/electrospinning), as well as integrated techniques. As a function of the used technique, this work aims to offer a comprehensive overview of the benefits/limitations of PCL-based scaffolds in order to establish a relationship between scaffold composition, namely integration of other biomaterial phases’ structural properties (i.e., pore morphology and mechanical properties) and in vivo response.

## 1. Introduction to Tissue Engineering

Tissue engineering (TE) emerged approximately 30 years ago as an alternative method for tissue and organ regeneration and restoration of function by implanting cells and biomolecules within a synthetic matrix named a scaffold. Furthermore, TE is a relatively new multidisciplinary field, which combines basic sciences such as materials science with biomechanics, cell biology, and medical sciences to repair, reconstruct, and restore the function of damaged or lost tissues and organs [1].

In different areas, bone defects show characteristic sizes, varying from millimeter recessions to complete resection of damaged tissue. Currently, the most commonly used methods to treat bone loss or damage are grafts (i.e., autografts, xenografts, allografts, isografts, and alloplastic grafts). Among them, autografts are the most widely used, despite showing several limitations, including multiple surgeries and the limited availability of tissue [2,3]. In this context, TE can be considered a valid therapeutic approach for surgery. Indeed, three-dimensional (3D) scaffolds working as an ECM template for cells firstly provide a 3D organization suitable for tissue growth and development. Moreover, they can be loaded with growth factors or biophysical stimuli able to direct growth and cell differentiation of cells toward tissue regeneration. In this context, recent technological advances open the possibility of creating even more reproducible scaffolds with the ability to be finely personalized in terms of structural/functional properties and customized as a function of the specific defect size, with relevant effects on future clinical practices [4,5]. Herein, we provide an accurate overview of current approaches on the design of PCL scaffolds, also emphasizing the biological implications of their use in animal models, offering an important survey to support translational research towards the definition of final trials for human patients.

## 2. Biomaterials: Basic Definitions and Classification

During the 1980s, biomaterials were defined at the Consensus Development Conference (Chester, UK, 1982) as “any substance, other than a drug, or a combination of substances, synthetic or natural in origin, which can be used for any period, as a whole or as a part of a system, which treats, augments or replaces any tissue, organ or function of the body” [6]. Hence, these materials are developed to regenerate tissues and organs in continuous contact with human body tissue cells. Additionally, biomaterials are classified as natural or synthetic, depending on their source of origin. Currently, different types of biomaterials are used to manufacture scaffolds, such as synthetic polymers, natural polymers, ceramics, metals, composites, and hydrogels, as shown in Table 1 [6,7].

### Why Poly (ε-Caprolactone)? Main Strengths and Weaknesses

Among the biomaterials currently used in the biomedical field, synthetic polymers are frequently preferred due to their workability that allows for the modulation of essential properties of 3D scaffolds to a large extent, including porosity, degradation, or mechanical properties, thus adapting them to medical application demands. Otherwise, they present overall drastically lower processing and storage costs compared to natural polymers, offering the opportunity to manipulate them in large quantities for the development of different products with high control of chemical/physical properties and durability as a function of the selected applicative target (i.e., different human body tissues) [1].

In this view, poly (ε-caprolactone) (PCL) has been considered one of the most promising polymers for biomedical applications in TE in the last three decades. It is a linear aliphatic polyester mainly obtained through ring-opening polymerization of ε-caprolactone monomers that can proceed through different polymerization mechanisms, including the cationic, anionic, coordination, or radical polymerization mechanisms [28]. All these reaction mechanisms can be strongly affected by several factors, such as polymerization duration and temperature, and the solution composition—i.e., monomer to solvent ratio or the presence/amount of catalysts, among others. By accurately controlling these parameters, it is possible to synthesize PCL with different molecular weights (Mn) and polydispersity indexes (PDIs) [30], characterized by different crystalline phases—usually not over 67%—and consequently different mechanical properties and chemical stability. All the scientific studies of PCL enabled the current production of different commercial PCL products with high quality, with controlled biodegradability and non-toxic properties recognized by the Food and Drug Administration (FDA). From the processing point of view, PCL shows peculiar characteristic temperatures—i.e., glass transition temperature (Tg) around −60 °C and melting point (Tm) around 60 °C—that makes it a valuable polymer suitable for biomedical applications [31]. One of the main weaknesses of PCL concerns its inability to be processed in the presence of aqueous solutions due to its strong hydrophobicity. However, several studies have widely demonstrated that PCL chains can be variously chemically functionalized to promote the formation of active sites able to confer the hydrophilic properties so relevant to improve specific cell interaction mechanisms (i.e., adhesion) [32].

Another important aspect is chemical stability and degradation. It is universally recognized that PCL shows the lowest degradation rate in vivo if compared to other biodegradable polymers used for biomedical applications (i.e., PLGA, PLA, etc. (Table 2)). In particular, several studies have verified that degradation approximately occurs from 2 to 3 years in a biological environment, by the formation of products easily excreted through the kidneys with no accumulation in the body [33].

Being part of the polyester class, PCL is susceptible to degradation mechanisms driven by hydrolysis occurring via a non-enzymatic, random chain scission catalyzed by acid and base reactions. In this context, PCL chain end-groups (i.e., hydroxyl groups) tend to be more stable than carboxylic acids, usually present in other polymers, thus contributing to enhancing the chemical stability of polymer chains in the presence of radicals or enzymatic activity [34]. In this view, PCL also shows interesting mechanical properties if compared with other biodegradable polyesters, offering the opportunity to support, to some extent, the loads that are typical of several tissues including cartilage, osteochondral, and porous bone [35]. Accordingly, several studies have demonstrated that PCL scaffolds are able to sustain and retain tissue properties after implantation until the full restoration of new tissue functions [36]. However, significant differences in the mechanical response can be highlighted as a function of chemical (i.e., molecular weight, degree of crystallinity, and chemical modification) or physical (i.e., pore size, porosity, interconnectivity) features, so that a wide set of PCL scaffolds—manufactured by different techniques—can be preferentially addressed for specific tissue targets—from soft to hard tissues.

Among the limitations of using PCL for clinical purposes is its slow degradation rate [37], which can range from 2 to 3 years [38], with some authors mentioning up to 5 years (which may not align with the rate of new bone tissue formation) [39], depending on the initial molecular weight and implant architecture. This slow degradation may not be ideal for all clinical applications, such as those requiring faster material resorption [38], and may also lead to complications such as scaffold encapsulation, chronic inflammation, or reduced adaptability for faster-healing environments.

Regarding its mechanical properties, such as compressive strength and elasticity, PCL may not be sufficient to support loads in high-stress areas of the human body. This limitation can restrict its use in applications requiring greater mechanical strength [38,40].

Pure PCL is a polymer with high hydrophobicity, which can negatively impact cellular interaction [39,41], delay osteogenic differentiation, and affect affinity [42] and integration with surrounding tissues [37,40,43]. To improve biocompatibility, PCL must be modified by adding bioactive materials [38].

It is important to note that there is insufficient scientific evidence supporting the use of PCL in clinical applications, and more clinical trials are needed to confirm the clinical utility of scaffolds in treating bone diseases [44]. These limitations suggest that while PCL has promising potential in regenerative medicine, modifications and combinations with other materials are required to optimize its performance in clinical settings.

**Table 2:** Advantages and disadvantages of different synthetic polymers used in tissue engineering.

	Advantages	Disadvantages	Ref
**Polycaprolactone (PCL)**	Biodegradable Longer degradation rate (2–3 years) Easy processing High strength High elongation Biocompatibility Flexible Less acidic degradation products	Hydrophobicity Poor cell adhesion Low melting point Soluble in organic solvents	[45,46,47]
**Poly(lactic acid) (PLA)**	Biocompatibility Biodegradable Stiffness Degrades faster than PCL Suitable mechanical properties	Poor solubility in organic solvents High sensitivity towards hydrolysis Low melting point Poor cell adhesion More acidic degradation products than PCL	[48,49,50]
**Poly(lactic-co-glycolic acid) (PLGA)**	Biocompability Biodegradable Eco-friendly Easy processing Excellent mechanical properties Controllable degradability	Hydrophobicity Lack of cell affinity Acidic degradation products Soluble in organic solvents	[51,52,53]

## 3. Structural Requirements for Scaffold Design

**Figure 1:**
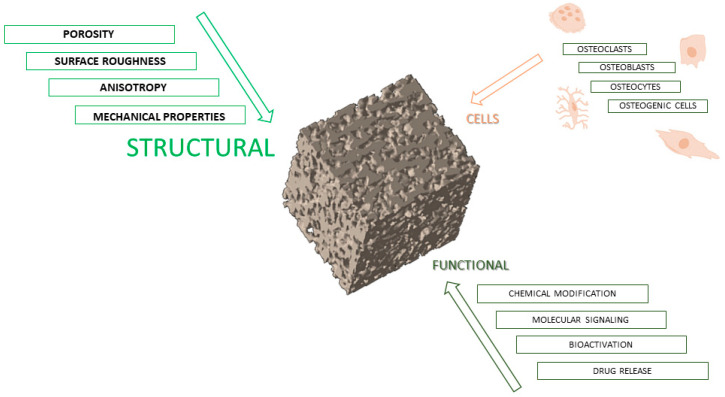
Schematic illustration of main elements for scaffold design in bone regeneration.

The essential features for bone scaffold applications must be understood from the native extracellular matrix tissue. If the morphological structure of the bone matrix can be biomimetically copied by a scaffold via any method and synthesized from any material, cellular response and regeneration can be achieved. Some essential features of scaffolds related to porosity, pore diameter, and roughness will be discussed and can be observed in Figure 1.

### 3.1. Porosity

Porosity is a predominant structural characteristic that a scaffold must have for regenerating bone tissue. This characteristic depends on the volume, size, shape, orientation, and interconnectivity between the scaffold pores. Additionally, it helps cell migration and proliferation by providing a suitable environment for transferring nutrients to the structures adjacent to the scaffold. Therefore, porous scaffolds uniformly enable cell growth, vascularization, and cell–cell distribution. Currently, the average scaffold porosity for regenerating bone tissue is between 50% and 90%, and the pore size is between 150 µm and 600 µm, depending on the type of bone to be repaired [36,54].

### 3.2. Pore Size

Pore size is certainly a crucial parameter in the design of 3D scaffolds for bone regeneration. It is known that osteoblasts with an average size ranging between 10 and 50 μm require adequate space to promote bone regeneration mechanisms [55]. Several experimental pieces of evidence have confirmed that small pores (<100 μm) tend to promote the formation of non-mineralized osteoid or fibrous tissue [56], while sufficiently large pores—with diameters larger than 100 μm—can more efficiently support the infiltration of macrophages and other cells involved in the different biological events—from colonization and migration to in vivo vascularization [57]. However, to date, the definition of an optimal pore size for bone regeneration is still highly controversial [58]. First pioneer studies demonstrated significant bone formation in the presence of pores greater than 800 μm. In this case, the smaller pores were predominantly colonized by fibroblasts, while the bone cells localized in larger pores, approximately 800 μm in diameter, were adequate to support cell growth [59]. Subsequently, other studies demonstrated that pores with a diameter between 250 and 400 μm, in the presence of bioactive signals, were able to stimulate the formation of mature bone, mainly promoting vascularization. Indeed, the formation of new vessels ensured an effective exchange of oxygen and nutrients that was required to support osteoblastic activity, as confirmed by the upregulation of osteopontin (OPN) and type I collagen [60].

More recently, other studies reported that pore sizes between 200 and 500 μm were optimal for osteoblast proliferation, without negatively affecting cell attachment [61]. Due to the implementation of new manufacturing methodologies, current scaffolds can be characterized by two distinct dimensional populations of pores: (a) smaller pores (<10 μm called micropores) offer a particularly high surface area able to stimulate greater ion exchange and protein adsorption bone [62]; (b) bigger pores with average diameters ranging from 100 to 325 μm provide an optimal architecture to promote cell proliferation [63]. In the presence of similar scaffolds, other studies have also highlighted the ability of scaffolds with larger pores to generate exogenous hypoxic conditions capable of stimulating the proliferation of endothelial cells [49], also inducing the secretion of proinflammatory cytokines—such as tumor necrosis factor α and interleukins 6, 10, 12, and 13—capable of triggering the bone regeneration response [64].

### 3.3. Pore Interconnectivity

The Standard Test Method for Microbial Ranking of Porous Packaging Materials (ASTM) uses terminology to describe porous materials. It classifies these materials into three groups: interconnecting pores (open pores), non-connecting (closed pores), and a combination of both. It is relevant to mention this because, in porous scaffolds, we can find open pores, closed pores, and pore interconnectivity. Therefore, open pores are connected orifices like tunnels reachable by gas, liquid, and particulate suspensions. However, closed pores are isolated pores that have no connection with the scaffold surface, do not provide for the cell microenvironment, and affect the mechanical properties of the scaffold. Also, we can find pore interconnectivity in the scaffolds, defined as the pore channels connecting different pores and establishing the scaffold permeability. This physical property is pivotal in scaffolds for bone tissue engineering because porous materials are critical in the tissue regeneration process and construction in the tissue engineering field. The literature mentions that the porous structure of the materials has a decisive role in determining the properties of the scaffolds since it can directly or indirectly affect the mechanical and biological scaffold [65,66,67].

### 3.4. Mechanical Properties

It is universally recognized that pore morphology is a key factor in the design of porous scaffolds. Besides its influence over the mechanisms of cell interaction and molecular transport, pore morphology plays a relevant role in particularizing the mechanical behavior of 3D scaffolds, with significant effects on biological response [68]. In the last two decades, several studies have investigated the strict correlation between mechanical response and cell behavior, depending on cell phenotype, scaffold chemical/physical properties, and micro-environmental conditions [69]. From the scaffold side, the mechanical properties have to be sufficient to avoid pore collapse during the regeneration process, ensuring fast recovery of the patient’s normal activities. In this view, the use of chemically stable polymers like PCL is mandatory to ensure specific features in terms of stiffness and strength suitable to achieve certain levels of mechanical performance required to properly interface structural tissues like bone during the regeneration process. The challenge consists of manipulating PCL using tailored manufacturing techniques to reach a good balance between porosity and mechanical response, finding the processing strategies to optimize the mechanical behavior for different porosity fractions as a function of the density of tissue to be replaced [70]. In the case of hierarchically organized tissue like bone, scaffolds also have to respect the complex architecture of the natural tissue, characterized by density gradients and anisotropic properties that confer unique properties in terms of lightness, stiffness, elasticity, and self-regeneration capabilities [71]. For instance, compact bone shows strength equal to 78.8–151 MPa (tensile) and 131–224 MPa (compression) in the longitudinal direction and 51–56 MPa (tensile)/106–133 MPa (compressive) in the transverse one. Conversely, cancellous bone—slightly anisotropic but highly porous—shows a strength of 5–10 MPa [72]. Overall, dense polymers are able to match cancellous bone properties, whereas bio ceramics show mechanical properties similar to those of cortical bone. As a function of the porosity degree, porous scaffolds show decay in mechanical properties about of one order of magnitude greater than cancellous bone and several orders of magnitude greater than cortical bone [73].

### 3.5. Surface Roughness

Scaffold surface roughness is an important property to consider when designing scaffolds for tissue regeneration, as this property has been shown to affect cell adhesion and proliferation. Therefore, scaffold surface roughness directly impacts cell morphology, proliferation, and phenotypic expression in vivo and in vitro studies. Depending on the extent of scaffold surface irregularity, surface roughness is divided into macro-roughness (100 µm—millimeters), micro-roughness (100 nm–100 µm), and nano-roughness (less than 100 nm) [59,74,75,76]. Several studies have shown that cells proliferate and grow on scaffolds with rough surfaces compared with scaffolds with smooth surfaces [56,77]. One of them is the study by Han et al., in which they fabricated porous 3D PCL scaffolds for bone tissue regeneration by selective laser sintering (SLS), evaluating various parameters of the SLS process that affected the roughness and biocompatibility of the scaffolds. For example, researchers found that scaffolds with a lower energy density during the SLS process improved scaffold roughness, indicating a positive effect on cell adhesion and proliferation [78].

## 4. Fabrication Methods for Bone Tissue Regeneration and In Vivo Implications

**Figure 2:**
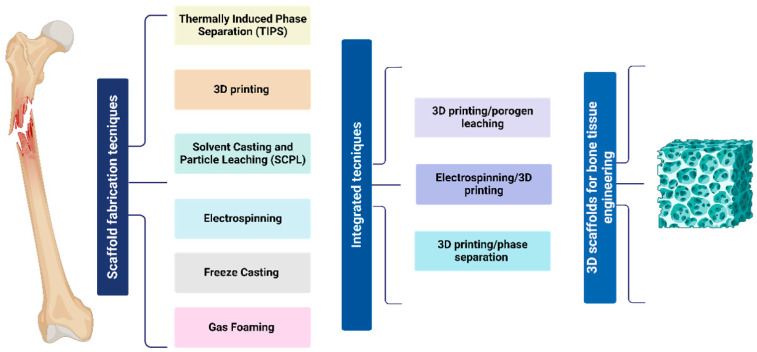
Schematic representation of specific scaffolding techniques for bone tissue.

There are several methods for fabricating PCL scaffolds for bone tissue engineering. Among them are the conventional methods and rapid prototyping (RP), which allow us to obtain scaffolds with different characteristics. Traditional methods for fabricating PCL scaffolds include particle leaching and solvent casting, thermally induced phase separation, freeze-drying, gas foaming, electrospinning, and sol–gel techniques. Meanwhile, rapid prototyping methods include stereolithography, 3D printing, fused deposition molding, and selective laser sintering. Additionally, integrated techniques can be used by combining two different manufacturing methods to gain advantages, thereby creating an optimal scaffold for tissue regeneration. All these different techniques allow us to obtain a porous morphological scaffolding for bone tissue, as shown in Figure 2.

### 4.1. Freeze Casting

Freeze casting, also called lyophilization or freeze drying, is a fabrication method for porous scaffolds for different tissue regeneration, including bone tissue. This manufacturing technique is made from various ceramic and polymeric materials and is suspended in a freezing vehicle. Subsequently, this suspension is frozen under certain conditions, followed by the freezing conductor’s sublimation, resulting in a porous scaffold formation. The synthesized scaffold depends on the thermal properties of the principal material that give specific anisotropic macroporous characteristics with homogeneous pore size and porosity throughout the scaffold structure. Some advantages of this method of manufacturing porous scaffolds are their low cost; the wide range of materials to be implemented in; its relatively simple manufacturing process; and its size, shape, direction, and length adaptability. Thus, the oriented microstructure obtained can benefit the final properties of the scaffold, i.e., mechanical, physicochemical, and biological behavior [1,79].

Several reports have shown that this manufacturing technique produces a structure with unidirectional pores that can improve water absorption and cellular behavior. For example, Ghorbani et al. demonstrated that manufactured PCL scaffolds using freeze-casting technology obtained a porous microstructure with open pores. Furthermore, when functionalized with polydopamine (PDA), they showed that porous PCL scaffolds have an open pore structure aligned with unidirectional channels. The scaffold improved the mechanical properties and hydrophilicity, improving the transfer of water and nutrients, increasing cell viability and cell–cell material interactions, and, most importantly, enhancing cell differentiation with the presence of alkaline phosphatase activity and upregulated the biomineralization and bone tissue regeneration capacity [79].

In another study, PCL scaffolds using a combination of chitosan and gelatin with different weight percentages were fabricated by freeze-drying for bone tissue engineering. The synthesis results of these scaffolds showed a porous structure and a pore diameter size ranging from 23 µm to 183 µm, which could promote cell growth and proliferation. This demonstrates that PCL can be used with other biomaterials to fabricate a 3D porous bone tissue regeneration scaffold [30].

**Figure 3:**
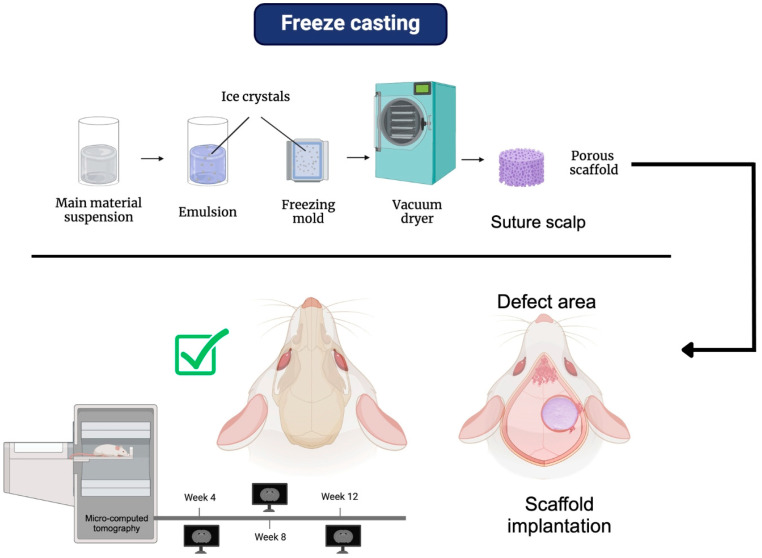
Scaffolds based on PCL via freeze casting and their biological response: Sprague Dawley rat calvaria defect model; evaluation of bone regeneration in defect area via micro-CT reconstruction images. Promising results were obtained.

Figure 3 shows a diagram of the freeze-casting process and some of its biological implications in an in vivo model. Various studies have shown that the fabrication of scaffolds using this technique produces highly porous 3D structures with an optimal pore size range of 75–250 µm, ideal for bone tissue regeneration [80]. It is important to note that the amount of solvent used in this technique can influence both porosity and average pore size [81]. Additionally, using two different polymers for scaffold fabrication, such as PCL and chitosan, has improved the scaffold’s contact angle, increasing its hydrophilicity, which is beneficial for tissue engineering applications [80].

In the study by Wang et al. (2020), in which 3D porous PCL scaffolds were fabricated using this technique with different amounts of 1,4-dioxane as a solvent to create a highly porous structure, they evaluated the in vivo biocompatibility and osteogenic capacity of the scaffolds in a calvarial defect model in Sprague Dawley rats. Eight weeks after surgery, X-ray images obtained through micro-CT showed higher densities in the critical-size defect for the PCL/1,4-dioxane groups (1:6, 1:8, 1:10, and 1:12 g/mL) compared to the control group. The color observed was similar to natural bone, indicating that the experimental groups had formed more new and mature bone tissue. Additionally, after 12 weeks of evaluation, a high density was observed, covering almost the entire critical-size defect area, with a color similar to the surrounding bone [81].

Furthermore, reconstructions obtained from micro-CT images revealed that, 8 weeks after surgery, there were larger sheets with high density similar to natural bone in the defects of the experimental groups. A similar result was seen at 12 weeks, with a notable increase in bone-like areas with high contrast [82]. A histological analysis showed that, after 12 weeks, both the region adjacent to the dura mater and the center of the scaffolds were occupied with bone-like tissue, and an apparent trabecular bone structure could be observed [82].

### 4.2. Gas Foaming

Gas foaming is a method that avoids using solvents; therefore, it is a better technique for incorporating sensitive molecules into scaffolds without reducing their bioactivity because of the use of carbon dioxide (CO_2_) or high-pressure nitrogen to infiltrate the polymer. In addition, these gasses have the characteristics of being non-toxic and non-flammable. This particularity gives gas-foaming advantages to synthesized scaffolds by incorporating growth factors, peptides, or bioactive molecules into the porous matrix size of approximately 100 µm with a porosity of 93%. However, the interconnectivity between the pores is low in the foam. So, to improve the interconnectivity between them, the particle leaching technique is required. Another disadvantage is that hydrophilic and glassy polymers cannot be used due to their low solubility in CO_2_ [1,82].

An example related to the manufacture of scaffolds using the gas foaming technique is the research carried out by Duarte et al., in which the authors evaluated the synthesis of 3D PCL scaffolds in a subcritical carbon dioxide atmosphere for the formation and delivery of an in situ foam associated with bone tissue regeneration. In this research, they synthesized foamed PCL scaffolds (45,000 Da) at 5.0 MPa and 45 °C with the best operating conditions. Therefore, it was possible to synthesize a porous 3D scaffold without supercritical conditions below 74 MPa [83]. In another research work, PCL and PCL/HA porous scaffolds were synthesized by the gas foaming technique, using supercritical carbon dioxide (scCO_2_) to investigate carvacrol impregnation as an antibacterial agent for bone tissue regeneration. In this research, the authors studied the effect of temperature, depressurization rate, hydroxyapatite (HA) content, and carvacrol impregnation on the changes in the scaffold morphology, porosity, and pore size. The results obtained were the synthesis of PCL porous scaffolds with carvacrol at 40 °C and 50 °C with different porosity values of 67% and 48%. Similar carvacrol loads of 7.71% and 7.22% by weight showed that the type of decompression rules the porosity of the scaffolds. Due to a second rapid decompression in the simultaneous impregnation and foaming experiments, the PCL scaffolds with carvacrol synthesized at 50 °C significantly decreased in porosity percentage and the size of the pores compared to the non-impregnated scaffolds. However, PCL/HA scaffolds with carvacrol synthesized at a temperature of 50 °C showed porosity values independent of carvacrol and HA content. Additionally, the PCL/HA scaffolds with carvacrol showed a higher rate of 10.24% and 10.57% by weight compared to the pure PCL scaffold with 7.22% by weight. The samples had a porosity percentage ranging between 47.69% and 49.07%, which confirmed that the porosity of the PCL scaffolds relies on the second decompression [84].

**Figure 4:**
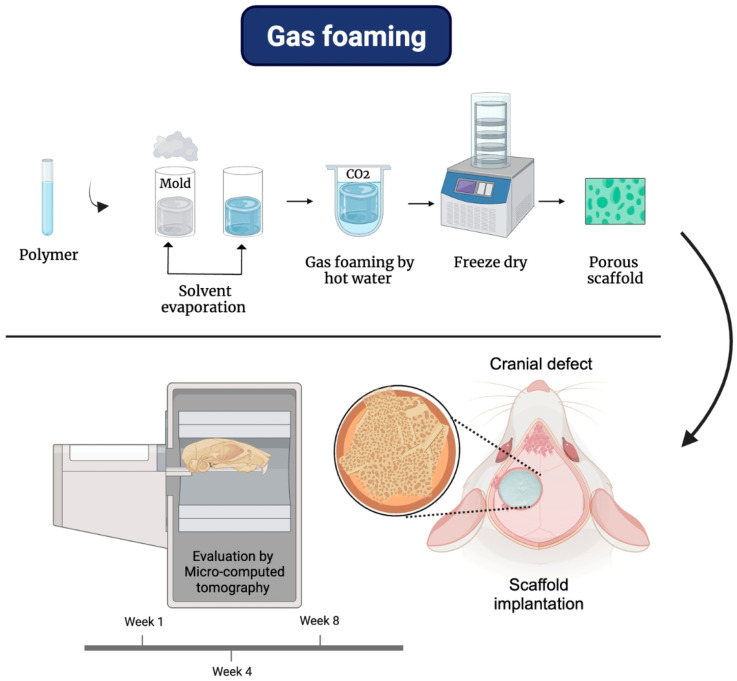
Scaffolds based on PCL via gas foaming and their biological response: evaluation of the osteogenic capacity of scaffolds in a rat cranial defect model. Micro-CT reconstruction images of the defect area for bone evaluation regeneration.

Figure 4 shows a schematic of the process for fabricating 3D scaffolds using the gas foaming technique and the biological implications of the scaffolds in an in vivo model. In the study conducted by Lou et al. (2022), a programmable porous polyurethane scaffold based on PCL with shape memory effect was fabricated using gas foaming, incorporating amorphous calcium phosphate modified with citrate (CAP) in different concentrations. The scaffolds obtained were named PCLCAP0, PCLCAP10, PCLCAP20, PCLCAP30, and PCLCAP40, where the number following “CAP” represents the weight percentage of CAP [85].

The PCL scaffolds doped with CAP exhibited a porous and interconnected surface morphology. Additionally, the porosity of the scaffolds gradually decreased with increasing CAP content. The contact angle results indicated that the PCLCAP40 scaffold showed increased hydrophilicity, attributed to the hydroxyl groups present in CAP. Through elemental mapping with EDS, it was determined that the calcium (Ca) and phosphorus (P) present on the surface of the scaffolds originated from the formation of hydroxyapatite, whose density increased with the CAP content. Furthermore, deposits found in PCLCAP20 significantly increased compared to PCLCAP0 [85].

The viability and proliferation of bone marrow-derived mesenchymal stem cells (BM-MSC) were evaluated using the CCK-8 assay, where the PCLCAP20 scaffold showed higher optical density (OD) values compared to PCLCAP0, suggesting that PCLCAP20 was beneficial for promoting cell proliferation [85].

In the in vivo evaluation using a rat cranial defect model for bone repair, it was observed that after one week of implantation, micro-CT images showed the presence of reparative tissue at the defect site, as well as bone-like apatite, which deposited on the scaffold surface due to the mineralization process. After 8 weeks, the PCLCAP20 scaffolds exhibited a more significant increase in new bone area than PCLCAP0. Moreover, bone volume (BV), bone volume fraction (BV/TV), trabecular number (Tb.N), and trabecular thickness (Tb.Th) were also higher in PCLCAP20 compared to the control, indicating that CAP was beneficial for bone tissue regeneration [85].

### 4.3. Solvent Casting and Particle Leaching (SCPL)

Particle leaching in combination with solvent casting represents a well-established, easy-to-reproduce, highly practicable, and low-cost technique for the fabrication of scaffolds. It allows for the creation of 3D porous structures through the removal of porogen agents—generally compounds soluble in water like sodium chloride, sugar, or gelatin—while the interconnections between the pores are guaranteed by the formation of micropores generated by the removal of the solvent. This method involves mixing porogen agents into a polymer solution in organic solvents not miscible in water. The mixture is poured into a mold and the solvent removed by forced or natural evaporation. Alternatively, the solvent can also be removed by the action of other water-soluble compounds, like ethanol, that is a non-solvent for the polymer used (phase inversion) [86]. Finally, the porogen particles are slowly leached in water until they completely dissolve the porogen agents and obtain a porous structure. Overall, this method allows for the control of porosity quite accurately—into a range from 50 to 90%—related to the volume fraction of the porogen agent used. Oppositely, this method shows some limits in the control of the size of the pores, often influenced by shrinkage phenomena occurring to the polymer matrix during the process.

This methodology is preferentially used with synthetic polymers such as polyethylene glycol (PEG), poly (lactic acid) (PLA), poly (glycolic acid) (PGA), and polycaprolactone (PCL) that allow for greater control of the process conditions with respect to natural ones, due to the high tunability of their microstructural properties (i.e., molecular weight). In particular, synthetic polymers like PCL with high molecular weight can offer a greater mechanical response, making them potentially suitable for applications in bone tissue [86].

**Figure 5:**
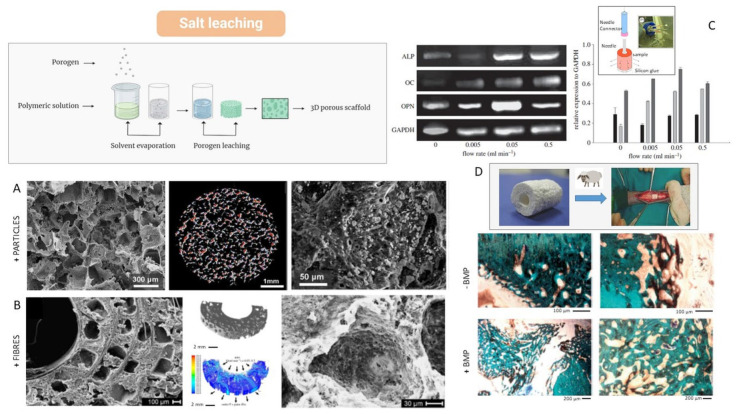
Scaffolds based on PCL via solvent casting and particle leaching and their biological response: (**A**) PCL scaffolds with HA solid signals—SEM images and 3D image analysis of pore architectures (red dots are HA particles with higher density) [63]; (**B**) PCL scaffolds reinforced with PLA long fibers [93]—SEM images and modeling based on computational fluid dynamics (CFD) simulation (92); (**C**) gene expression by RT-PCR of relevant bone-related markers (ALP (black), OC (light grey), and OPN (dark grey)) and quantitative estimation of signal for different flow rates (in the square, the perfusion system used) [92]; (**D**) histological evaluation in a femoral defect sheep model at 12 weeks with/without BMP entrapment [94].

Sempertegui et al. proposed microporous PCL scaffolds when varying different manufacturing parameters to evaluate the mechanical properties, microstructure, water absorption capacity, and cellular response. They demonstrated that structural homogeneity was promoted by the use of larger NaCl porogen particles (106–425 µm vs. 63–106 µm). An increase in pore size improves cell colonization, despite the surface-to-volume ratio, without negatively influencing the mechanical properties [87]. Another study proposed the use of the salt leaching technique for the creation of porous PCL scaffolds with graphene additives. In this case, the scaffolds characterized by interconnected porosity also showed an increase in mechanical and degradation properties, suggesting potential use for bone tissue regeneration. In fact, the addition of a targeted quantity of graphene increased the hydrophilicity of the surfaces, favoring the cellular response without any evident cytotoxic response [88]. Alternatively, PCL may also be added to peculiar inorganic phases like calcium phosphates, which, in addition to providing an effective bioactive signal, can positively influence the biomechanical response of the scaffold [89,90] (Figure 5A). In particular, Guarino et al. demonstrated that the integration of magnesium carbonate and hydroxyapatite into PCL porous scaffolds induces the formation of ectopic bone tissue in CD-1 nu/nu mice, thus confirming the osteoinductive and osteoconductive capability of the scaffold to promote new bone formation in a biological environment [63].

By applying the basic principles of continuous fiber-reinforced composites to the scaffold design, Guarino et al. have also proposed a porous scaffold based on the integration of resorbable PLA fibers by the filament winding technique, coupled with the conventional salt leaching technique [91] (Figure 5B). In this case, degradation preferentially occurs at the fiber–polymer interface, resulting in a higher rate of degradation than for either material alone. In this case, the helicoidal organization of continuous fibers also ensures a strong mechanical interlocking between the fiber and matrix, thus minimizing the breakdown occurring at the interface (Figure 5C). Noteworthily, the biological behavior of these composite scaffolds depends upon the presence of a multi-scale porosity with tailored characteristics in terms of pore interconnections and pore size as a consequence of the peculiar degradation mechanisms of single phases. Hence, the well-organized pore network within the scaffold may potentially control cell colonization and fluid transport through its peculiar geometry [92], with the combined effects of the reinforcing action of PLLA fibers and the slow degradation rate of the PCL matrix undoubtedly contributing to the biological response in osteogenic way (Figure 5C). Then, changes in fiber and matrix features, such as diameter and fiber chemistry, during degradation could provide a guide for the bone remodeling process by achieving a composite structure with morphological and structural properties, which then evolve, during degradation, towards a softer and more porous material needed in the long term. A further improvement is obtained by the preparation of fibrous scaffolds incorporating bioactive rigid particles and thus combining the reinforcement action of bioactive phases (i.e., hydroxyapatite) with the tailored degradation kinetics of biodegradable and/or resorbable polymers [93]. In this case, scaffolds both mimic the composition and structural organization of natural bone tissue, giving the opportunity to support the in vivo regeneration of bone tissue also in large defects (Figure 5D) [94] in which, at 12 weeks, the scaffolds did not cause inflammation or foreign body reactions. The scaffolds without BMP limited new bone formation to the edges, while the scaffolds with BMP promoted greater bone formation, even in the internal area of the scaffold [94].

### 4.4. Thermally Induced Phase Separation (TIPS)

Thermally induced phase separation is a simple and versatile technique for the fabrication of porous scaffolds from binary polymer solutions. The basic idea provides the cooling of a polymer solution to induce a separation in two different phases, respectively, one polymer-rich phase and another polymer-poor phase. Later, the removal of the solvent within the polymer-poor phase by solvent evaporation or sublimation allows for reaching an open pores network whereas the polymer which composes the polymer rich phase solidifies in the final structure of the scaffold [95].

This process is regulated by a complex process depending on the thermodynamic and the kinetic mechanisms occurring during the cooling process [73].

**Figure 6:**
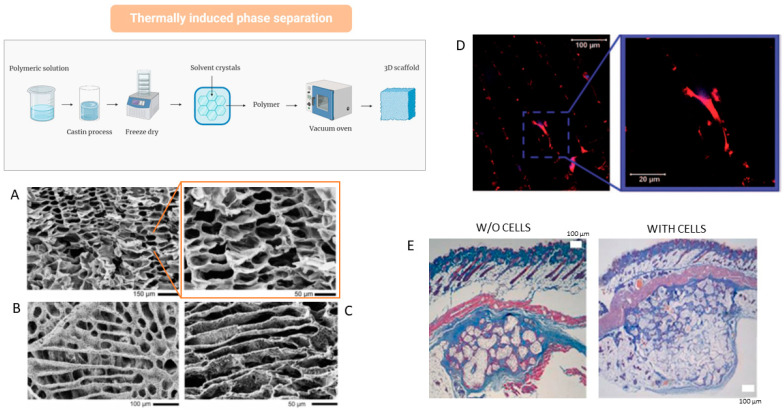
Scaffolds based on PCL via thermally induced phase separation: (**A**) scanning electron microscopy (SEM) images of PCL scaffolds obtained via the TIPS/solvent extraction technique: cross-sectional SEM images at low (scale bar: 150 mm) and high (scale bar: 50 mm) magnification; (**B**,**C**) different pore morphologies due to different solvent extraction mechanisms namely, PCL/DMSO (**B**) (scale bar: 100 mm) and PCL/Dioxane (**C**) binary systems [95]; (**D**) confocal images of actin microfilaments (in red) and nuclei (in blue) of hMSCs grown on porous PCL scaffolds [97]; (**E**) PCL scaffolds implanted subcutaneously in the back of athymic nude mice: histological images and Masson’s trichrome or HE staining. Scale bar (100 μm) [98].

When the fixed temperature is higher than the solvent crystallization one or freezing point, liquid–liquid phase separation usually occurs; differently, solid–liquid phase separation takes place when the solvent crystallization temperature overcomes the cooling one [96]. Phase diagram is the tool usually used to investigate the behavior of binary solutions—namely high-molecular-weight polymers plus low-molecular-weight solvents—under a controlled thermal history (Figure 6).

At high temperatures, the binary system is homogeneous and stable by forming only one phase. Moving down inside the bimodal curve, the formation of two phases occurs—a polymer-rich phase and a polymer-poor one—through two mechanisms of nucleation and growth or spinodal decomposition as a function of the solution concentration [99]. In particular, they drastically affect the morphology of pores varying from closed pore structures—falling into the binodal/spinodal inter-regions—until fully interconnected pore networks—inside the spinodal regions.

In this view, TIPS can be considered a valid technique to widely modulate pore morphology by varying process parameters (i.e., temperature and cooling rate) and relative polymer and solvent concentration, giving the opportunity to change, consequently, other structural properties (i.e., fluid transport, molecular release, and mechanical properties).

For instance, TIPS allows for the control of pore anisotropy, depending on the solvent choice and phase separation conditions (Figure 6A–C). The ability to adjust the size and interconnectivity of these pores within the scaffolds enhances fluid access to the porous internal structure, facilitating the diffusion of nutrients and molecular factors.

Interestingly, pore networks with peculiar morphological features (i.e., aligned pores) may also provide a morphological guide for cell adhesion (Figure 6D), proliferation, and phenotypic expression [95,97]. Nevertheless, the main limitations concern the use of polymer solutions with well-defined viscosities, restricting its use to a few polymers, its molecular weight, and/or its solution concentrations [100]. Another intrinsic constraint of TIPS is the ability to form pore networks with characteristic sizes over 100 microns that are not optimal to support bone cell adhesion and proliferation [95].

Moreover, in order to overcome some intrinsic limitations of polymers in terms of cell interactions, dispersing inorganic particles or biological macromolecules are also provided in the polymer matrix to endow bioactive signals able to mimic the biomechanical properties of the mineralized extracellular matrix [95]. However, no relevant studies are reported in the literature to exclusively evaluate TIPS scaffolds in animal models but the majority of them involve the use of TIPS in combination with other manufacturing techniques. For instance, Milian et al. proposed to combine salt leaching and TIPS to fabricate porous PCL scaffolds embedded with alginate to support osteochondral regeneration in vitro and in vivo. It is demonstrated that alginate improved the chondrogenic activity in vitro by increasing the expression of markers related to osteo/chondrogenesis, also supporting vascularization in vivo (Figure 6E).

Samadian et al. developed a bioactive 3D scaffold by dispersing Taurine-loaded gelatin electrospun nanofibers into a polylactic acid (PLA) and polycaprolactone (PCL) solution [101]. They obtained highly porous scaffolds—porosity > 90%—with optimal in vitro response in terms of cell proliferation for cell growth, as well as in vivo response in terms of newly formed bone tissue, due to the synergic effect of the fully interconnected porous network and Taurine release on bone signal regulation and metabolism [101].

Similar studies investigated the effect of nanoclays in 3D porous scaffolds of polycaprolactone/gelatin clay (PCL/GNF/NC) loaded with different doses of like silybin (Sil) [102]. These scaffolds with porosity ranging from 70% to 90% showed improved mechanical properties, due to the presence of nanoclays, and improved in vitro cell response in terms of cell viability ascribable to the effect of silybin while preliminary in vivo results suggested a promising use for the treatment of bone defects [102].

### 4.5. Electrospinning

Electrospinning is a simple technique that allows for the creation of micro- and nanofibers through a jet of synthetic or natural polymeric solutions, which is electrically charged or through a polymer in a molten state. However, a combination of both materials is required due to the poor stability of natural polymers and the toxicity of synthetic polymer products.

**Figure 7:**
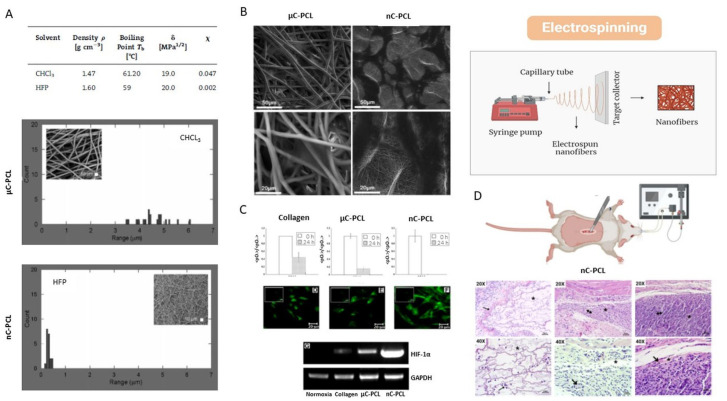
Scaffolds based on PCL via electrospinning leaching and their biological response: (**A**) fiber diameter distribution via image analysis of electrospun fibers from chloroform (μC-PCL) and HFP (nC-PCL) solutions (SEM images of different PCL fibers in the square) [104]; (**B**) FESEM images of adherent MSC after 24 h to estimate the effect of micro/nanostructure of PCL fibers on the in vitro response of hMSC [104]; (**C**) effect- of micro and nanofibrous texture in collagen sandwich on hypoxic conditions via HIF_1a expression and PCR analysis (C is control of fibrillary collagen) N [105]; (**D**) in vivo study of PCL nanofibers in a rat model: H&E staining images (20× and 40×) after 1, 2, and 3 weeks after implantation (arrows indicate neo-forming small-caliber vessels at 10 days of implantation) [106].

The electrospinning system consists of four components: a syringe pump, a metal needle, a high-voltage power supply, and a grounded connector. This technique involves pumping a polymeric solution through the tip of a needle placed on the opposite side of the grounded collector. Therefore, the jet of liquid spreads is formed through electrostatic repulsion expansion loops until deposited on the grounded collector. Consequently, the fibers produced by this technique are called electrospun fibers, which can create scaffolds that mimic the native extracellular matrix of the body and allow cell adhesion, proliferation, and development. As a result, with this technique, we obtain a three-dimensional (3D) network of fibers, which can generate a porous and interconnected surface (Figure 7). This can be controlled by modifying the polymer concentration, solvent properties and the electrospinning device adjustment. 

This technique—based on the controlled solvent evaporation—is a simple and fast method for 3D porous scaffold manufacturing, in comparison with other approaches based on fiber extrusion/solidification (i.e., melt spinning/electrospinning) [103]. otherwise, a relevant one disadvantage is related to a non-homogeneous distribution of the pores [1,92].

In vitro studies have shown that topographic signals exerted by PCL scaffolds with different fiber diameters about from 500 nm to 5 µm drastically influence cell infiltration, survival rate, proliferation (Figure 7B) [104], also affecting oxygen metabolism (Figure 7C) [105], and the deposition of mineralized phases. Lastly, in vivo studies confirmed the ability of on 3D PCL fibrous scaffold with the appropriate morphological and biochemical cues to promote a suitable osteoblast environment by supporting the formation of small-caliber vessels just after 10 days of implantation (Figure 7D) [106].

In another study, PCL electrospun fibrous scaffolds, functionalized with gelatin and with a high or low degree of anisotropy, were synthesized to investigate how the fiber orientation of the scaffold affects the behavior of osteogenic cells. The results showed that PCL fibers could control the morphology and orientation of mature osteoblasts. They also observed that aligned fibers could promote osteogenesis and higher mineral deposition compared to scaffolds with random fibers. A similar result was obtained when they used human embryonic stem cell-derived mesenchymal progenitor cells (hES-MPs) because both types of cells were oriented along the main direction of fiber alignment. Likewise, in both cell types, the scaffolds with aligned fibers exhibited a higher degree of organization of the mineralized matrix, which increased the stiffness of the scaffolds. However, hES-MP differentiation was better in scaffolds with unaligned fibers. Therefore, the study reveals the complex relationship between external directional signals and osteogenic differentiation [107] (Figure 7D).

### 4.6. 3D Printing

Additive manufacturing (AM) is a processing method that guarantees complete control over the scaffold microarchitecture and shape, allowing good reproducibility. This method is ideal for manufacturing complex scaffolds without using other tools or molds. Among the processing methods of additive manufacturing (AM) are Selective Laser Sintering (SLS), Fusion Extrusion or Fused Deposition Modeling (FDM), and Three-Dimensional Printing (3DP). All these techniques consist of obtaining a composition layer by layer from a three-dimensional computer-aided design (CAD) model, which achieves high precision, resolution, and reproducibility. An advantage of the scaffolds produced by AM is that they have a wholly controlled structure. They also have a porous network of more than 90%, which is interconnected, and they are entirely reproducible. One of its disadvantages is that there is no currently defined standard design for the design of bone scaffolds since the scaffold design will depend on the area of the defect to be repaired [108,109].

Some studies have used three-dimensional (3D) printing technology for scaffold manufacturing for bone tissue regeneration. One of them is the research by Wang et al., in which they synthesized PCL/HA composite filaments for material extrusion applications. Therefore, they produced PCL/HA scaffolds (HA 0%, 5%, 10%, 15%, 20%, and 25% *w*/*w* PCL) and analyzed the fabrication parameters. Within the results, they found that the porosity and pore size of the different groups of scaffolds were similar, and all showed interconnected structures. Furthermore, higher HA increased the surface roughness and scaffolds’ hydrophilicity compared to the PCL scaffolds. Also, the increase in HA improved the elastic modulus and the resistance to compression. Consequently, filaments composed of PCL with different proportions of HA were fabricated to subsequently print 3D scaffolds, which have the necessary characteristics for bone tissue regeneration [110].

In another study, scientists used a simple melt-mix approach to make polycaprolactone/bioglass granules to synthesize 3D scaffolds with a pore size gradient. This research used an additive manufacturing system based on screw-assisted extrusion for PCL-bioglass scaffold fabrication for bone tissue regeneration. In the results, the researchers obtained that the diameter of the filament decreased as the ceramic content increased, generating an increase in the pore size. These results could explain the increase in melt viscosity due to adding bioglass. Furthermore, adding bioglass particles did not show significant differences in the hydrophilic properties of the scaffolds. Also, it was possible to notice that bioglass improved scaffolds’ mechanical properties, thus mimicking cortical bone’s mechanical properties. Likewise, from day 7 to day 14, a significant increase in cellular metabolic activity was observed in the bioglass scaffolds. Also, the results showed that the contents of calcium, oxygen, and phosphorus increased with the bioglass content since it has been reported that bioglass acts as a nucleation agent. Consequently, the pore size gradient also induced a cell density gradient, with a higher number of cells in the outer region of the scaffold and a lower number in the inner region [111].

**Figure 8:**
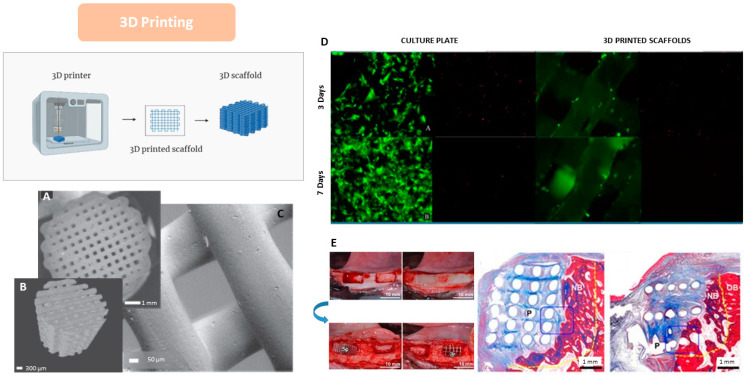
Scaffolds based on PCL via 3D printing and their biological response: (**A**) 2D optical image, (**B**) 3D micro-CT reconstruction, (**C**) topographic image by secondary electrons source [112]; (**D**) live/dead staining assay using calcein-AM (live in green) and ethidium homodimer-1 (dead in red) to estimate viability and DPSC proliferation. Results showed a higher proportion of live than dead cells on PCL 3D-printed scaffolds than on the control (culture plate), all images 40× [113]; (**E**) histology examination of bone regeneration after PCL scaffold implantation. Histomorphometric analysis showed the presence of newly formed bone (NB, new bone; OB, old bone; P, PCL scaffold) [114].

The PCL scaffolds observed in Figure 8A–C were synthesized using a bioplotter device, which deposited 3D fibers to fabricate the scaffold. Figure 8A corresponds to a 2D optical image, while Figure 8B shows images obtained through micro-CT, revealing the scaffolds’ pore size, microstructure, and interconnected network. Figure 8C shows a structure with high control over pore geometry and fiber surface roughness [112].

In another study, 3D-printed PCL scaffolds were fabricated and seeded with dental pulp stem cells (DPSCs) derived from porcine tissue to evaluate the viability, proliferation, differentiation, and mineralization of the cells in the scaffolds (Figure 8D). The results of the live-cell and adhesion assays on the 3D-printed PCL scaffolds demonstrated the differentiation between live/dead cells and the adhesion of DPSCs in the PCL scaffolds [113]. Live cell fluorescence appears green in Figure 8D, while dead cells appear bright red after 3 and 7 days of incubation. The results of this assay showed a higher number of live cells compared to dead cells in the 3D-printed PCL scaffolds compared to the control [113].

Additionally, in the research by Park et al. (2018), the in vivo implications of the 3D PCL scaffolds were evaluated, and the effectiveness of a customized 3D PCL scaffold fabricated with a bioprinting system to facilitate alveolar bone regeneration was investigated. As shown in Figure 8E, saddle-shaped bone defects were created in the mandibles of Beagle dogs, where PCL scaffolds with different strand sizes were placed in the defects, and β-TCP powder particles were applied to stimulate bone growth around the defects. The strand thickness/period for the two groups was 400/400 and 400/1200 µm. The experimental samples were divided into three groups: the scaffold containing PCL blocks and β-TCP powder as a control, the scaffold with 400/400 networks with β-TCP powder, and the scaffold with 400/1200 networks with β-TCP powder. The results obtained by micro-CT after the in vivo experiments showed a higher rate of bone formation in the 400/1200 group compared to the particle group. The histological images in Figure 8E showed that the new bone formation was consistent with the results found in the micro-CT, and new bone formation was observed above the bottom of the defects in all groups; in contrast, the vertical dimensions of the defects were well maintained in the 400/400 and 400/1200 block groups [114].

## 5. Integrated Techniques

In the previous paragraph, different scaffold fabrication techniques were discussed, highlighting their benefits in forming a pore network suitable to support bone regeneration. For instance, the use of porogens in the salt leaching technique allows for the control of the porosity degree by the used fraction of leachable particles as well as the pore size by using particles of different sizes. Meanwhile, the obtained porous structure tends to present intact pore walls with only a few interconnection points, so generating a closed cellular structure with several limitations in terms of cell colonization and molecular transport. In this case, the use of other techniques like phase inversion/separation can help to form a secondary population of smaller pores able to create the interconnections among the bigger ones. More in general, the use of integrated manufacturing techniques can help to design more detailed multiscale porous architectures able to reproduce the complexity of the structural/functional properties of the extracellular matrix of the natural tissue, especially in the case of hierarchically organized tissues like bone.

In this section, different approaches based on the integration of different manufacturing techniques for the fabrication of PCL scaffolds will be introduced by remarking on the major advantages in terms of morphological/structural properties and their in vivo implications.

### 5.1. 3D Printing/Porogen Leaching

**Figure 9:**
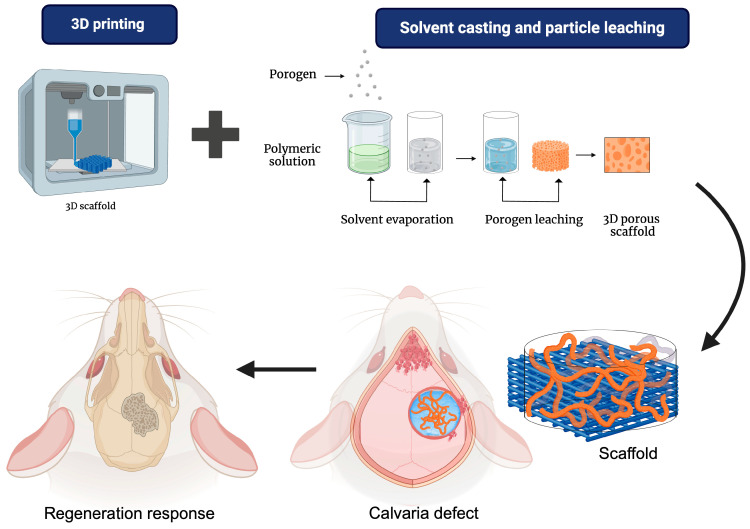
Scaffolds based on PCL via 3D printing and solvent casting and particle leaching and their biological response in a rat calvaria defect model for the evaluation of bone regeneration.

In the study by Dang et al. 2020, 3D printing techniques were combined with particle leaching to develop scaffolds with dual-scale porosity. Additionally, their capacity for guided bone regeneration was evaluated in a critical-size calvarial defect model in rats, as seen in Figure 9. The scaffolds were additively manufactured using medical-grade PCL doped with porogen microparticles to create microscale porosity. The groups formed were nmPCL (non-porous mPCL scaffold), pmPCL17 (porous mPCL scaffold with 17% porogen), and pmPCL44 (porous mPCL scaffold with 44% porogen). According to the microscale porosity, SEM images showed pore size and surface geometry differences. Smaller pores were observed in the pmPCL scaffolds due to the leaching of the porogen agent. However, all groups had no statistically significant differences in the surface pore areas [115].

On the other hand, in the dual-scale porous groups, it was demonstrated that pmPCL44 scaffolds had larger intra-punctual microscale pores and greater porosity compared to pmPCL17. Furthermore, in the in vitro evaluation, the pmPCL44 scaffolds showed more excellent cell adhesion than the other groups. Additionally, in vivo studies in a critical-size calvarial defect in rats showed that pmPCL44 scaffolds promoted new bone formation [115]. In other studies, biological outcomes are partially conflicting, showing a predominance of inflammatory phenomena and foreign body responses on the formation of new bone tissue. Despite 3D printing being universally recognized as a gold standard for shape and pore size reproducibility, limited control of the surface properties and mechanical response could be ascribable to the combination with traditional techniques (i.e., salt leaching or phase separation), with negative effects on in vitro and in vivo response.

### 5.2. 3D Printing/Phase Separation

The combination of techniques for fabricating PCL scaffolds shows promise. Creating a scaffold with 3D printing using layer-by-layer fused deposition provides three-dimensional solid support. Additionally, using thermally induced phase separation aims to produce a nanometric-level porous structure, which increases the available surface for cell adhesion and proliferation while mimicking the morphology of bone tissue [116].

**Figure 10:**
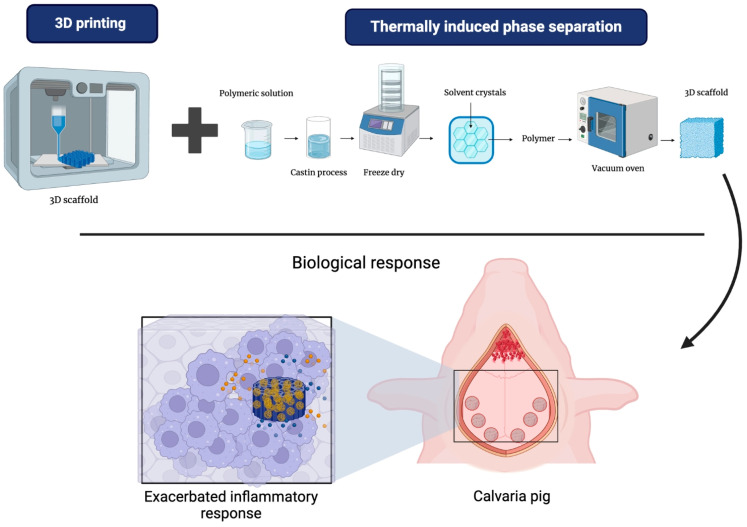
Scaffolds based on PCL via 3D printing and thermally induced phase separation and their biological response in a pig calvaria defect model and their inflammatory response.

The integrated scaffold was tested on a cranial defect in Landrace pigs, as seen in Figure 10. The results demonstrated an unfavorable response due to the abundant infiltration of multinucleated giant cells, indicative of a foreign body-type inflammatory response [116].

Despite the theoretical advantages of this scaffold, which was fabricated by combining these techniques, the results in the animal model did not show a favorable response. This suggests that, although combining these two techniques may offer design benefits, the biological response in a living environment may be more complex and require further optimization.

### 5.3. 3D Printing/Electrospinning

The use of 3D-printed scaffolds enables the creation of customized, complex structures, while electrospinning adds a layer of nanofibers that provide flexibility and porosity. These properties enhance cell interactions and improve the delivery of growth factors crucial for tissue regeneration. This combination strengthens the mechanical and biological properties of the scaffold, creating a structure that more closely mimics the native extracellular matrix. As a result, it promotes cell adhesion, proliferation, and differentiation, which ultimately supports tissue regeneration [117].

**Figure 11:**
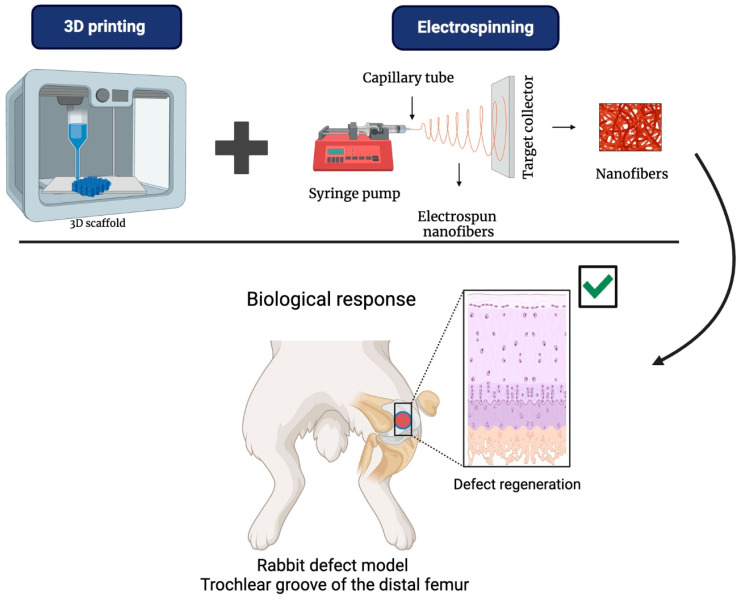
Scaffolds based on PCL via 3D printing and electrospinning and their biological response in a rabbit distal femoral bone defect model and the defect regeneration.

In vivo studies have examined the response of these scaffolds. Jie Liu and colleagues developed a two-layer hybrid scaffold, as seen in Figure 11. The top layer comprises a PCL/gel nanofiber membrane conjugated with heparin and prepared using electrospinning. This layer serves as a barrier, preventing the invasion of fibrous connective tissue into the bone defect. The bottom layer is 3D-printed using a PCL/Gel/n-HA (PGH) scaffold, designed to facilitate bone tissue regeneration and support the upper membrane, preventing collapse. The scaffold was tested in an osteochondral defect model in the knees of New Zealand rabbits. After 20 weeks, the study showed that the defect sites treated with the hybrid scaffold exhibited a significantly higher degree of new bone formation than the control group [118]. These findings suggest that the hybrid scaffold holds excellent potential for clinical applications in hard and soft tissue regeneration [118].

## 6. Conclusions and Future Trends

PCL is one of the most currently used polymers to manufacture 3D porous scaffolds for bone tissue regeneration, due to its peculiar properties in terms of biocompatibility, mechanical properties, and long degradation times. Its high processability also allows us to adapt a wide set of manufacturing technologies—from the most traditional to the newest ones—for the production of a plethora of 3D scaffolds with different morphological and structural properties that are suitable for supporting bone regeneration. This is crucial to provide more accurate in vitro experimental studies in subsequent years to better understand cell materials’ interaction mechanisms in more detail, progressively overcoming the lack of standardization still present in the definition of optimal scaffold properties (i.e., porosity and pore sizes) for bone regeneration.

Another important target concerns the biomimetic properties of PCL scaffolds. Despite PCL being universally recognized as biocompatible, it needs to be combined with bioactive particles (i.e., calcium phosphates, bioglasses, nanotubes, and graphene/mxenes) and/or biomacromolecules (i.e., proteins, growth factors, peptides, amino acids, lipids, and flavonoids) to impart osteoinductive/osteoconductive cues able to more efficiently support bone healing and immunomodulatory effects. Recent studies have demonstrated the ability of calcium phosphate/PCL nanocomposite scaffolds to promote mineralization and support bone regeneration for 2 years during in vivo implantation in critical-sized pig cranial models [119]. However, more studies are still needed, and preclinical/clinical investigations are required to reach approval of these bioactive biomaterials for use as a biomedical product, due to constraints in the development of accurate translational research.

Otherwise, further studies are still required to design and manufacture innovative electroactive biomaterials that are susceptive to the action of external forces like ultrasound, electrical and/or magnetic fields—potentially able to facilitate bone regeneration through functional rehabilitation of the natural electro-physiological microenvironment [120]. To date, all the mechanisms involving the role of electrical cues on bone healing are well known, while the effects of electroactive biomaterials on the biological response in terms of immunomodulation, angiogenesis, osteogenesis, and bone remodeling are still under investigation. In the near future, an important challenge will be to implement tailored manufacturing techniques suitable for efficiently mediating electrical cues to bone without negatively affecting other key scaffold properties (i.e., mechanical properties and degradation) that are crucial in guiding in vitro and in vivo bone regeneration.

Meanwhile, the road to clinical use is still long, and the fabrication of biomedical products suitable for clinical translation from bench to bedside still needs a bit of time. Indeed, most scaffold manufacturing techniques—presented in this review—are not still ready for clinical trials for different reasons—e.g., lack of reproducibility in structural/functional properties and limits on personalization (i.e., geometry and defect anatomy). Among them, bioprinting is certainly the manufacturing technique more accredited for rapid clinical translation. The high precision in controlling porosity and pore size, as well as in placing growth factors and/or cells into a preordered architecture, is essential to promote efficient scaffold integration with bone tissue. Accordingly, some examples of 3D-printing scaffolds for bone regeneration have recently been released onto the market (Ossiform™, Cerabone^®^), showing positive outcomes from clinical practice. However, this is only a first step toward minimally invasive in vivo scaffolds addressing immediate defect repair and the definition of personalized models for accurate disease modeling and drug screening.

## Figures and Tables

**Table 1:** Multiple biomaterials employed in tissue engineering.

Biomaterials	Advantages	Disadvantages	Examples	Product Name	Applications	Refs
**Natural Polymers**	Promote cell adhesion Biocompatibility Bioactivity Enhance cellular activity	Weak mechanical properties Complex processing Not reproducible in large quantities Uncontrollable degradation rate	Collagen types I and III Agarose and alginate Silk	Mucograft Cartipatch^®^ Silk voice^®^	Gigival recession Knee cartilage injury Wound healing	[7,8,9,10,11]
**Synthetic Polymers**	Mimics the structural and mechanical properties of the MEC Low cost Easy processing Bioinert Produced in large quantities Tunable mechanical properties	Use toxic solvents for its preparation Low biocompatibility Deficient bioactivity	Polyglycolic acid (PGA), self-reinforced (SR) Poly-l-lactic acid 70% (PLLA), poly-d,l-lactic acid 30% (PDLLA) Poly-l-lactic acid 82% (PLLA), polyglycolic acid 18% (PGA)	Biofix^®^ SR MacroPore^®^ Lactosorb^®^	Maxillofacial bone surgery Bone fixation Maxillofacial bone surgery	[7,10,12,13,14,15,16,17]
**Bioceramics**	Biocompatibility Moderate degradation Osteoinductivity High mechanical Stiffness High melting temperatures	Low elasticity Brittleness Extremely high stiffness Slow degradation Low toughness Poor fatigue resistance	Calciumphospho-silicate bioactive glass (Bioglass 45S5) Calcium phospho-silicate bioactive glass (Bioglass 45S5) Coralline hydroxyapatite (HA)	BonAlive^®^ Perioglas^®^ Bio-Eye^®^	Orthopedic and maxillofacial reconstruction Maxillofacial defects Orbital implants	[7,16,17,18,19,20,21]
**Metals**	Good mechanical properties Better yield strength Better fatigue strength High elastic modulus High degrees of ductility Biocompatibility	Reduced cell adhesion to their surface Can release toxic metallic ions or particles Corrosive action Lack of tissue adherence	Stainless steel Ti85Zr15 alloy Tantalum	Prestige ST^®^ Roxolid^®^ Trabecular Metal™ Technology	Cervical disc replacement Dental prothesis Femoral head necrosis	[7,22,23,24,25,26,27]
**Composites**	Combination of different materials Controllable mechanical properties such as stiffness, strength, and toughness Biocompatibility Optimized properties	Complex processing	Polyglycolic acid (PGA), hydroxyapatite (HA) Cobalt–chromium platform scaffolds containing sirolimus biodegradable polymers Fibrinogen; poly (lactide-co-epsilon-caprolactone)	Chondrotissue^®^ GenoDES TM Chongshu^®^ composite hernia patch	Cartilage tissue engineering Coronary stent implantation Hernia repair	[7,11,28,29]

## References

[1] Eltom A., Zhong G., Muhammad A. (2019). Scaffold Techniques and Designs in Tissue Engineering Functions and Purposes: A Review. Adv. Mater. Sci. Eng..

[2] Dang M., Saunders L., Niu X., Fan Y., Ma P.X. (2018). Biomimetic delivery of signals for bone tissue engineering. Bone Res..

[3] Wei H., Cui J., Lin K., Xie J., Wang X. (2022). Recent advances in smart stimuli-responsive biomaterials for bone therapeutics and regeneration. Bone Res..

[4] Rosales Ibáñez R., Alvarado Estrada K.N., Ojeda Gutiérrez F. (2012). Ingeniería Tisular en Odontología. Rev. Adm..

[5] Murphy C.M., O’brien F.J., Little D.G., Schindeler A. (2013). Cell-scaffold interactions in the bone tissue engineering triad. Eur. Cell Mater..

[6] Dolcimascolo A., Calabrese G., Conoci S., Parenti R. (2019). Innovative Biomaterials for Tissue Engineering. Biomaterial-Supported Tissue Reconstruction or Regeneration.

[7] Kesharwani R.K., Keservani R.K., Sharma A.K. (2022). Tissue Engineering.

[8] Donnaloja F., Jacchetti E., Soncini M., Raimondi M.T. (2020). Natural and Synthetic Polymers for Bone Scaffolds Optimization. Polymers.

[9] Puertas-Bartolomé M., Mora-Boza A., García-Fernández L. (2021). Emerging Biofabrication Techniques: A Review on Natural Poly-mers for Biomedical Applications. Polymers.

[10] Han F., Wang J., Ding L., Hu Y., Li W., Yuan Z., Guo Q., Zhu C., Yu L., Wang H. (2020). Tissue Engineering and Regenerative Medicine: Achievements, Future, and Sustainability in Asia. Front. Bioeng. Biotechnol..

[11] Chen M., Jiang R., Deng N., Zhao X., Li X., Guo C. (2022). Natural polymer-based scaffolds for soft tissue repair. Front. Bioeng. Biotechnol..

[12] Phutane P., Telange D., Agrawal S., Gunde M., Kotkar K., Pethe A. (2023). Biofunctionalization and Applications of Polymeric Nanofibers in Tissue Engineering and Regenerative Medicine. Polymers.

[13] Williams D.F. (2019). Challenges With the Development of Biomaterials for Sustainable Tissue Engineering. Front. Bioeng. Biotechnol..

[14] On S.-W., Cho S.-W., Byun S.-H., Yang B.-E. (2020). Bioabsorbable Osteofixation Materials for Maxillofacial Bone Surgery: A Review on Polymers and Magnesium-Based Materials. Biomedicines.

[15] Al-Shalawi F.D., Ariff A.H.M., Jung D.-W., Ariffin M.K.A.M., Kim C.L.S., Brabazon D., Al-Osaimi M.O. (2023). Biomaterials as Implants in the Orthopedic Field for Regenerative Medicine: Metal versus Synthetic Polymers. Polymers.

[16] Baino F., Hamzehlou S., Kargozar S. (2018). Bioactive Glasses: Where Are We and Where Are We Going?. J. Funct. Biomater..

[17] Baino F. (2016). Bioceramics and Composites for Orbital Implants: Current Trends and Clinical Performance. Handbook of Bioceramics and Biocomposites.

[18] Pina S., Reis R.L., Oliveira J.M. (2018). Ceramic biomaterials for tissue engineering. Fundamental Biomaterials: Ceramics.

[19] Baino F., Novajra G., Vitale-Brovarone C. (2015). Bioceramics and Scaffolds: A Winning Combination for Tissue Engineering. Front. Bioeng. Biotechnol..

[20] Pawan K., Brijnandan S.D., Anil S. (2018). Bioceramics for Hard Tissue Engineering Applications: A Review. Int.-Natl. J. Appl. Eng. Res..

[21] Tanvir A.H., Khaleque A., Kim G.-H., Yoo W.-Y., Kim Y.-Y. (2024). The Role of Bioceramics for Bone Regeneration: History, Mechanisms, and Future Perspectives. Biomimetics.

[22] Lv Y., Wang B., Liu G., Tang Y., Lu E., Xie K., Lan C., Liu J., Qin Z., Wang L. (2021). Metal Material, Properties and Design Methods of Porous Biomedical Scaffolds for Additive Manufacturing: A Review. Front. Bioeng. Biotechnol..

[23] Chowdhury S.K., Nagarjuna V., Bhaskar B. (2021). Metallic Biomaterials in Tissue Engineering: Retrospect and Prospects. Bio-Materials in Tissue Engineering and Regenerative Medicine.

[24] Qi J., Yu T., Hu B., Wu H., Ouyang H. (2021). Current Biomaterial-Based Bone Tissue Engineering and Translational Medicine. Int. J. Mol. Sci..

[25] Liu Z., Guo W., Li Z., Cheng L., Zhang Q., Yue D., Shi Z., Wang B., Sun W., Zhang N. (2014). Porous tantalum rods for treating osteonecrosis of the femoral head. Genet. Mol. Res..

[26] Pham M.H., Mehta V.A., Tuchman A., Hsieh P.C. (2015). Material Science in Cervical Total Disc Replacement. BioMed Res. Int..

[27] Berlanga-Acosta J., Fernández-Montequín J., Valdés-Pérez C., Savigne-Gutiérrez W., Mendoza-Marí Y., García-Ojalvo A., Falcón-Cama V., del Barco-Herrera D.G., Fernández-Mayola M., Pérez-Saad H. (2017). Mechanical Charac-terisation and Biomechanical and Biological Behaviours of Ti-Zr Binary-Alloy Dental Implants. BioMed Res. Int..

[28] Sultana N., Hassan M.I., Lim M.M. (2015). Composite Synthetic Scaffolds for Tissue Engineering and Regenerative Medicine.

[29] Elena S., Claudio M. (2003). Composite materials for biomedical applications: A review. J. Appl. Biomater. Biomech..

[30] Malikmammadov E., Tanir T.E., Kiziltay A., Hasirci V., Hasirci N. (2018). PCL and PCL-based materials in biomedical applications. J. Biomater. Sci. Polym. Ed..

[31] Gupta D., Dogra V., Verma D., Chaudhary A.K., Tewari M. (2024). PCL-based composites and their utilizations in the medical sector. Bioresorbable Polymers and Their Composites.

[32] Sowmya B., Hemavathi A.B., Panda P.K. (2021). Poly (ε-caprolactone)-based electrospun nano-featured substrate for tissue engineering applications: A review. Prog. Biomater..

[33] Mondal D., Griffith M., Venkatraman S.S. (2016). Polycaprolactone-based biomaterials for tissue engineering and drug delivery: Current scenario and challenges. Int. J. Polym. Mater. Polym. Biomater..

[34] Ouhadi T., Stevens C., Teyssié P. (1976). Study of poly-ε-caprolactone bulk degradation. J. Appl. Polym. Sci..

[35] Liu G., Wei X., Zhai Y., Zhang J., Li J., Zhao Z., Guan T., Zhao D. (2024). 3D printed osteochondral scaffolds: Design strategies, present applications and future perspectives. Front. Bioeng. Biotechnol..

[36] Prasadh S., Wong R.C.W. (2018). Unraveling the mechanical strength of biomaterials used as a bone scaffold in oral and maxillofacial defects. Oral Sci. Int..

[37] Deshpande M.V., Girase A., King M.W. (2023). Degradation of Poly(ε-caprolactone) Resorbable Multifilament Yarn under Physiological Conditions. Polymers.

[38] Javkhlan Z., Hsu S.-H., Chen R.-S., Chen M.-H. (2024). 3D-printed polycaprolactone scaffolds coated with beta tricalcium phosphate for bone regeneration. J. Formos. Med. Assoc..

[39] Janmohammadi M., Nourbakhsh M.S., Bahraminasab M., Tayebi L. (2023). Effect of Pore Characteristics and Alkali Treatment on the Physicochemical and Biological Properties of a 3D-Printed Polycaprolactone Bone Scaffold. ACS Omega.

[40] Dwivedi R., Kumar S., Pandey R., Mahajan A., Nandana D., Katti D.S., Mehrotra D. (2020). Polycaprolactone as biomaterial for bone scaffolds: Review of literature. J. Oral Biol. Craniofacial Res..

[41] Nyberg E., Rindone A., Dorafshar A., Grayson W.L. (2017). Comparison of 3D-Printed Poly-ε-Caprolactone Scaffolds Functionalized with Tricalcium Phosphate, Hydroxyapatite, Bio-Oss, or Decellularized Bone Matrix. Tissue Eng. Part A.

[42] Liang H.-Y., Lee W.-K., Hsu J.-T., Shih J.-Y., Ma T.-L., Vo T.T.T., Lee C.-W., Cheng M.-T., Lee I.-T. (2024). Polycaprolactone in Bone Tissue Engineering: A Comprehensive Review of Innovations in Scaffold Fabrication and Surface Modifications. J. Funct. Biomater..

[43] Park S.H., Park S.A., Kang Y.G., Shin J.W., Park Y.S., Gu S.R., Wu Y.R., Wei J., Shin J.-W. (2017). PCL/β-TCP Composite Scaffolds Exhibit Positive Osteogenic Differentiation with Mechanical Stimulation. Tissue Eng. Regen. Med..

[44] Re F., Borsani E., Rezzani R., Sartore L., Russo D. (2023). Bone Regeneration Using Mesenchymal Stromal Cells and Biocompatible Scaffolds: A Concise Review of the Current Clinical Trials. Gels.

[45] Asaduzzaman F., Salmon S. (2023). Controllable Water-Triggered Degradation of PCL Solution-Blown Nanofibrous Webs Made Possible by Lipase Enzyme Entrapment. Fibers.

[46] MacCraith E., O’brien F., Davis N. (2021). Biodegradable materials for surgical management of stress urinary incontinence: A narrative review. Eur. J. Obstet. Gynecol. Reprod. Biol..

[47] Ilyas R.A., Zuhri M.Y.M., Norrrahim M.N.F., Misenan M.S.M., Jenol M.A., Samsudin S.A., Nurazzi N.M., Asyraf M.R.M., Supian A.B.M., Bangar S.P. (2022). Natural Fiber-Reinforced Polycaprolactone Green and Hybrid Biocomposites for Various Advanced Applications. Polymers.

[48] Lopes M.S., Jardini A., Filho R.M. (2012). Poly (Lactic Acid) Production for Tissue Engineering Applications. Procedia Eng..

[49] Englert C., Brendel J.C., Majdanski T.C., Yildirim T., Schubert S., Gottschaldt M., Windhab N., Schubert U.S. (2018). Pharmapolymers in the 21st century: Syn-thetic polymers in drug delivery applications. Prog. Polym. Sci..

[50] Łysik D., Deptuła P., Chmielewska S., Bucki R., Mystkowska J. (2022). Degradation of Polylactide and Polycaprolactone as a Result of Biofilm Formation Assessed under Experimental Conditions Simulating the Oral Cavity Environment. Materials.

[51] Makadia H.K., Siegel S.J. (2011). Poly Lactic-co-Glycolic Acid (PLGA) as Biodegradable Controlled Drug Delivery Carrier. Polymers.

[52] Gentile P., Chiono V., Carmagnola I., Hatton P.V. (2014). An Overview of Poly(lactic-*co*-glycolic) Acid (PLGA)-Based Biomaterials for Bone Tissue Engineering. Int. J. Mol. Sci..

[53] Guzmán-Soria A., Moreno-Serna V., Canales D.A., García-Herrera C., Zapata P.A., Orihuela P.A. (2023). Effect of Electrospun PLGA/Collagen Scaffolds on Cell Adhesion, Viability, and Collagen Release: Potential Applications in Tissue Engineering. Polymers.

[54] Turnbull G., Clarke J., Picard F., Riches P., Jia L., Han F., Li B., Shu W. (2018). 3D bioactive composite scaffolds for bone tissue engineering. Bioact. Mater..

[55] Sugawara Y., Kamioka H., Honjo T., Tezuka K.-I., Takanoyamamoto T. (2005). Three-dimensional reconstruction of chick calvarial osteocytes and their cell processes using confocal microscopy. Bone.

[56] Wang C., Xu D., Lin L., Li S., Hou W., He Y., Sheng L., Yi C., Zhang X., Li H. (2021). Large-pore-size Ti6Al4V scaffolds with different pore structures for vascularized bone regeneration. Mater. Sci. Eng. C.

[57] Iviglia G., Kargozar S., Baino F. (2019). Biomaterials, Current Strategies, and Novel Nano-Technological Approaches for Periodontal Regeneration. J. Funct. Biomater..

[58] Mohammadi H., Sepantafar M., Muhamad N., Bakar Sulong A. (2021). How Does Scaffold Porosity Conduct Bone Tissue Regeneration?. Adv. Eng. Mater..

[59] Chang H.-I., Wang Y. (2011). Cell Responses to Surface and Architecture of Tissue Engineering Scaffolds. Regenerative Medicine and Tissue Engineering—Cells and Biomaterials.

[60] Cheng M.-Q., Wahafu T., Jiang G.-F., Liu W., Qiao Y.-Q., Peng X.-C., Cheng T., Zhang X.-L., He G., Liu X.-Y. (2016). A novel open-porous magnesium scaffold with controllable microstructures and properties for bone regeneration. Sci. Rep..

[61] Lim T.C., Chian K.S., Leong K.F. (2010). Cryogenic prototyping of chitosan scaffolds with controlled micro and macro architecture and their effect on in vivo neo-vascularization and cellular infiltration. J. Biomed. Mater. Res. Part A.

[62] Morejón L., Delgado J.A., Ribeiro A.A., de Oliveira M.V., Mendizábal E., García I., Alfonso A., Poh P., van Griensven M., Balmayor E.R. (2019). Development, Characteri-zation and In Vitro Biological Properties of Scaffolds Fabricated From Calcium Phosphate Nanoparticles. Int. J. Mol. Sci..

[63] Guarino V., Scaglione S., Sandri M., Alvarez-Perez M.A., Tampieri A., Quarto R., Ambrosio L. (2014). MgCHA particles dispersion in porous PCL scaffolds: In vitro mineralization and in vivo bone formation. J. Tissue Eng. Regen. Med..

[64] Mukherjee S., Darzi S., Paul K., Werkmeister J.A., Gargett C.E. (2019). Mesenchymal stem cell-based bioengineered constructs: Foreign body response, cross-talk with macrophages and impact of biomaterial design strategies for pelvic floor disorders. Interface Focus.

[65] Reis R.L., Roma S. (2005). Biodegradable Systems in Tissue Engineering and Regenerative Medicine.

[66] Sauerova P., Suchy T., Supova M., Bartos M., Klima J., Juhasova J., Juhas S., Kubikova T., Tonar Z., Sedlacek R. (2019). Positive impact of dynamic seeding of mesenchymal stem cells on bone-like biodegradable scaffolds with increased content of calcium phosphate nanoparticles. Mol. Biol. Rep..

[67] Murphy C.M., O’Brien F.J. (2010). Understanding the effect of mean pore size on cell activity in collagen-glycosaminoglycan scaffolds. Cell Adh. Migr..

[68] Kumar A., Nune K.C., Murr L.E., Misra R.D.K. (2016). Biocompatibility and mechanical behaviour of three-dimensional scaffolds for biomedical devices: Process–structure–property paradigm. Int. Mater. Rev..

[69] Eshraghi S., Das S. (2010). Mechanical and microstructural properties of polycaprolactone scaffolds with one-dimensional, two-dimensional, and three-dimensional orthogonally oriented porous architectures produced by selective laser sintering. Acta Biomater..

[70] Vincenzo G., Filippo C., Luigi A. (2007). Porosity and Mechanical Properties Relationship in PCL Porous Scaffolds. Appl. Biomater. Biomech..

[71] Guarino V., Gloria A., Raucci M.G., De Santis R., Ambrosio L. (2012). Bio-inspired composite and cell instructive platforms for bone regeneration. Int. Mater. Rev..

[72] Yaszemski M.J., Payne R.G., Hayes W.C., Langer R., Mikos A.G. (1996). Evolution of bone transplantation: Molecular, cellular and tissue strategies to engineer human bone. Biomaterials.

[73] Velasco M.A., Narváez-Tovar C.A., Garzón-Alvarado D.A. (2015). Design, Materials, and Mechanobiology of Biodegradable Scaffolds for Bone Tissue Engineering. BioMed Res. Int..

[74] Chen H., Huang X., Zhang M., Damanik F., Baker M.B., Leferink A., Yuan H., Truckenmüller R., van Blitterswijk C., Moroni L. (2017). Tailoring surface nanoroughness of electrospun scaffolds for skeletal tissue engineering. Acta Biomater..

[75] Calore A.R., Srinivas V., Groenendijk L., Serafim A., Stancu I.C., Wilbers A., Leoné N., Sanchez A.A., Auhl D., Mota C. (2023). Manufacturing of scaffolds with interconnected internal open porosity and surface roughness. Acta Biomater..

[76] Sengupta P., Prasad B.L.V. (2018). Surface Modification of Polymers for Tissue Engineering Applications: Arginine Acts as a Sticky Protein Equivalent for Viable Cell Accommodation. ACS Omega.

[77] Deng Y., Liu X., Xu A., Wang L., Luo Z., Zheng Y., Deng F., Wei J., Tang Z., Wei S. (2015). Effect of surface roughness on osteogenesis in vitro and osseointegration in vivo of carbon fiber-reinforced polyetheretherketone-nanohydroxyapatite composite. Int. J. Nanomed..

[78] Han J., Li Z., Sun Y., Cheng F., Zhu L., Zhang Y., Zhang Z., Wu J., Wang J. (2022). Surface Roughness and Biocompatibility of Polycaprolactone Bone Scaffolds: An Energy-Density-Guided Parameter Optimization for Selective Laser Sintering. Front. Bioeng. Biotechnol..

[79] Ghorbani F., Zamanian A., Sahranavard M. (2020). Mussel-inspired polydopamine-mediated surface modification of freeze-cast poly (ε-caprolactone) scaffolds for bone tissue engineering applications. Biomed. Eng. Biomed. Technol..

[80] Lor Huai Chong Zarith N.Z., Sultana N. (2015). Poly(Caprolactone)/chitosan-based scaffold using freeze drying technique for bone tissue engineering application. Proceedings of the 2015 10th Asian Control Conference (ASCC).

[81] Wang S., Yang Y., Koons G.L., Mikos A.G., Qiu Z., Song T., Cui F., Wang X. (2020). Tuning pore features of mineralized collagen/PCL scaffolds for cranial bone regeneration in a rat model. Mater. Sci. Eng. C.

[82] Manoukian O.S., Sardashti N., Stedman T., Gailiunas K., Ojha A., Penalosa A., Mancuso C., Hobert M., Kumbar S.G. (2019). Biomaterials for Tissue Engineering and Regenerative Medicine. Encyclopedia of Biomedical Engineering.

[83] Duarte R.M., Correia-Pinto J., Reis R.L., Duarte A.R.C. (2018). Subcritical carbon dioxide foaming of polycaprolactone for bone tissue regeneration. J. Supercrit. Fluids.

[84] Satpayeva A., Rojas A., Tyrka M., Ksepko E., Galotto M.J., Zizovic I. (2022). Supercritical Foaming and Impregnation of Polycaprolactone and Polycaprolactone-Hydroxyapatite Composites with Carvacrol. Processes.

[85] Luo K., Wang L., Chen X., Zeng X., Zhou S., Zhang P., Li J. (2022). Biocompatible Poly(ε-caprolactone)-based Shape-memory Polyu-rethane Composite Scaffold with Bone-induced Activity. J. Bionic. Eng..

[86] Cho Y.S., Hong M.W., Quan M., Kim S., Lee S., Lee S., Kim Y.Y. (2017). Assessments for bone regeneration using the polycaprolactone SLUP (salt-leaching using powder) scaffold. J. Biomed. Mater. Res. Part A.

[87] Sempertegui N.D., Narkhede A.A., Thomas V., Rao S.S. (2018). A combined compression molding, heating, and leaching process for fabrication of micro-porous poly(ε-caprolactone) scaffolds. J. Biomater. Sci. Polym. Ed..

[88] Huang H.-Y., Fan F.-Y., Shen Y.-K., Wang C.-H., Huang Y.-T., Chern M.-J., Wang Y.-H., Wang L. (2020). 3D poly-ε-caprolactone/graphene porous scaffolds for bone tissue engineering. Colloids Surf. A Physicochem. Eng. Asp..

[89] Chocholata P., Kulda V., Babuska V. (2019). Fabrication of Scaffolds for Bone-Tissue Regeneration. Materials.

[90] Ruiz-Aguilar C., Olivares-Pinto U., Drew R.A.L., Aguilar-Reyes E.A., Alfonso I. (2020). Porogen Effect on Structural and Physical Prop-erties of β-TCP Scaffolds for Bone Tissue Regeneration. IRBM.

[91] Guarino V., Causa F., Taddei P., di Foggia M., Ciapetti G., Martini D., Fagnano C., Baldini N., Ambrosio L. (2008). Polylactic acid fibre-reinforced polycaprolactone scaffolds for bone tissue engineering. Biomaterials.

[92] Guarino V., Urciuolo F., Alvarez-Perez M.A., Mele B., Netti P.A., Ambrosio L. (2012). Osteogenic differentiation and mineralization in fibre-reinforced tubular scaffolds: Theoretical study and experimental evidences. J. R. Soc. Interface.

[93] Guarino V., Ambrosio L. (2008). The synergic effect of polylactide fiber and calcium phosphate particle reinforcement in poly ε-caprolactone-based composite scaffolds. Acta Biomater..

[94] Ronca A., Guarino V., Raucci M.G., Salamanna F., Martini L., Zeppetelli S., Fini M., Kon E., Filardo G., Marcacci M. (2014). Large defect-tailored composite scaffolds for in vivo bone regeneration. J. Biomater. Appl..

[95] Guarino V., Ambrosio L. (2010). Temperature-driven processing techniques for manufacturing fully interconnected porous scaffolds in bone tissue engineering. Proc. Inst. Mech. Eng. Part H J. Eng. Med..

[96] Akbarzadeh R., Yousefi A. (2014). Effects of processing parameters in thermally induced phase separation technique on porous ar-chitecture of scaffolds for bone tissue engineering. J. Biomed. Mater. Res. B Appl. Biomater..

[97] Guarino V., Guaccio A., Guarnieri D., Netti P.A., Ambrosio L. (2012). Binary system thermodynamics to control pore architecture of PCL scaffold via temperature-driven phase separation process. J. Biomater. Appl..

[98] Milián L., Oliver-Ferrándiz M., Peregrín I., Sancho-Tello M., Martín-De-Llano J.J., Martínez-Ramos C., Carda C., Mata M. (2024). Alginate Improves the Chondrogenic Capacity of 3D PCL Scaffolds In Vitro: A Histological Approach. Curr. Issues Mol. Biol..

[99] Guarino V., Causa F., Salerno A., Ambrosio L., Netti P.A. (2008). Design and manufacture of microporous polymeric materials with hierarchal complex structure for biomedical application. Mater. Sci. Technol..

[100] Ghalia M.A., Dahman Y. (2016). Advanced nanobiomaterials in tissue engineering: Synthesis, properties, and applications. Nanobiomaterials in Soft Tissue Engineering: Applications of Nanobiomaterials.

[101] Samadian H., Farzamfar S., Vaez A., Ehterami A., Bit A., Alam M., Goodarzi A., Darya G., Salehi M. (2020). A tailored polylactic acid/polycaprolactone biodegradable and bioactive 3D porous scaffold containing gelatin nanofibers and Taurine for bone regeneration. Sci. Rep..

[102] Tayeed M.H., Tehranchi M., Ehterami A., Shanei F., Taleghani F., Semyari H., Mehrnia N., Bozorgzadeh S., Salehi M. (2021). Bone Regeneration in Rat Using a PCL/gelatin/Nanoclay Nanocomposite Scaffold Containing Silybin. Res. Sq..

[103] Zaiss S., Brown T.D., Reichert J.C., Berner A. (2016). Poly(ε-caprolactone) Scaffolds Fabricated by Melt Electrospinning for Bone Tissue Engineering. Materials.

[104] Guarino V., Cirillo V., Taddei P., Alvarez-Perez M.A., Ambrosio L. (2011). Tuning Size Scale and Crystallinity of PCL Electrospun Fibres via Solvent Permittivity to Address hMSC Response. Macromol. Biosci..

[105] Guaccio A., Guarino V., Perez M.A.A., Cirillo V., Netti P.A., Ambrosio L. (2011). Influence of electrospun fiber mesh size on hMSC oxygen metabolism in 3D collagen matrices: Experimental and theoretical evidences. Biotechnol. Bioeng..

[106] Cruz-Maya I., Cirillo V., Serrano-Bello J., Serri C., Alvarez-Perez M.A., Guarino V. (2024). Optimization of Diclofenac-Loaded Bicom-ponent Nanofibers: Effect of Gelatin on In Vitro and In Vivo Response. Pharmaceutics.

[107] Delaine-Smith R.M., Hann A.J., Green N.H., Reilly G.C. (2021). Electrospun Fiber Alignment Guides Osteogenesis and Matrix Organi-zation Differentially in Two Different Osteogenic Cell Types. Front. Bioeng. Biotechnol..

[108] Madrid A.P.M., Vrech S.M., Sanchez M.A., Rodriguez A.P. (2019). Advances in additive manufacturing for bone tissue engineering scaffolds. Mater. Sci. Eng. C.

[109] Yang Y., Wang G., Liang H., Gao C., Peng S., Shen L., Shuai C. (2018). Additive manufacturing of bone scaffolds. Int. J. Bioprint..

[110] Wang F., Tankus E.B., Santarella F., Rohr N., Sharma N., Märtin S., Michalscheck M., Maintz M., Cao S., Thieringer F.M. (2022). Fabrication and Characterization of PCL/HA Filament as a 3D Printing Material Using Thermal Extrusion Technology for Bone Tissue Engineering. Polymers.

[111] Daskalakis E., Huang B., Vyas C., Acar A.A., Fallah A., Cooper G., Weightman A., Koc B., Blunn G., Bartolo P. (2022). Novel 3D Bioglass Scaffolds for Bone Tissue Regeneration. Polymers.

[112] Guarino V., Gloria A., De Santis R., Ambrosio L., Dumitriu S., Popa V. (2013). Manufacturing Multifunctional Scaffolds for Tissue Engineering. Polymeric Biomaterials.

[113] Rosales-Ibáñez R., Cubo-Mateo N., Rodríguez-Navarrete A., González-González A.M., Villamar-Duque T.E., Flores-Sánchez L.O., Rodríguez-Lorenzo L.M. (2021). Assessment of a PCL-3D Printing-Dental Pulp Stem Cells Triplet for Bone Engineering: An In Vitro Study. Polymers.

[114] Park S.A., Lee H.-J., Kim K.-S., Lee S.J., Lee J.-T., Kim S.-Y., Chang N.-H., Park S.-Y. (2018). In Vivo Evaluation of 3D-Printed Polycaprolactone Scaffold Implantation Combined with β-TCP Powder for Alveolar Bone Augmentation in a Beagle Defect Model. Materials.

[115] Dang H.P., Vaquette C., Shabab T., Pérez R.A., Yang Y., Dargaville T.R., Shafiee A., Tran P.A. (2020). Porous 3D Printed Scaffolds For Guided Bone Re-generation In a Rat Calvarial Defect Model. Appl. Mater. Today.

[116] Jensen J., Rölfing J.H.D., Le D.Q.S., Kristiansen A.A., Nygaard J.V., Hokland L.B., Bendtsen M., Kassem M., Lysdahl H., Bünger C.E. (2014). Surface-modified functionalized poly-caprolactone scaffolds for bone repair: In vitro and in vivo experiments. J. Biomed. Mater. Res. A.

[117] Yang D., Faraz F., Wang J., Radacsi N. (2022). Combination of 3D Printing and Electrospinning Techniques for Biofabrication (Adv. Mater. Technol. 7/2022). Adv. Mater. Technol..

[118] Liu J., Zou Q., Wang C., Lin M., Li Y., Zhang R., Li Y. (2021). Electrospinning and 3D printed hybrid bi-layer scaffold for guided bone regeneration. Mater. Des..

[119] Helaehil J.V., Lourenço C.B., Huang B., Helaehil L.V., de Camargo I.X., Chiarotto G.B., Santamaria M., Bártolo P., Caetano G.F. (2021). In Vivo Investigation of Poly-mer-Ceramic PCL/HA and PCL/β-TCP 3D Composite Scaffolds and Electrical Stimulation for Bone Regeneration. Polymers.

[120] Luo S., Zhang C., Xiong W., Song Y., Wang Q., Zhang H., Guo S., Yang S., Liu H. (2024). Advances in electroactive biomaterials: Through the lens of electrical stimulation promoting bone regeneration strategy. J. Orthop. Transl..

